# Therapeutic developments for SARS-CoV-2 infection—Molecular mechanisms of action of antivirals and strategies for mitigating resistance in emerging variants in clinical practice

**DOI:** 10.3389/fmicb.2023.1132501

**Published:** 2023-03-02

**Authors:** Oana Săndulescu, Cătălin Gabriel Apostolescu, Liliana Lucia Preoțescu, Adrian Streinu-Cercel, Mihai Săndulescu

**Affiliations:** ^1^Carol Davila University of Medicine and Pharmacy, Bucharest, Romania; ^2^National Institute for Infectious Diseases “Prof. Dr. Matei Balș”, Bucharest, Romania

**Keywords:** COVID-19, antivirals, neutralizing antibodies, monoclonal antibodies, therapeutic efficacy, resistance

## Abstract

This article systematically presents the current clinically significant therapeutic developments for the treatment of COVID-19 by providing an in-depth review of molecular mechanisms of action for SARS-CoV-2 antivirals and critically analyzing the potential targets that may allow the selection of resistant viral variants. Two main categories of agents can display antiviral activity: direct-acting antivirals, which act by inhibiting viral enzymes, and host-directed antivirals, which target host cell factors that are involved in steps of the viral life cycle. We discuss both these types of antivirals, highlighting the agents that have already been approved for treatment of COVID-19, and providing an overview of the main molecules that are currently in drug development. Direct-acting antivirals target viral enzymes that are essential in the viral life cycle. Three direct-acting antivirals are currently in use: two are nucleoside analogs that inhibit the RNA-dependent RNA polymerase of SARS-CoV-2, i.e., remdesivir and molnupiravir, and the third one, nirmatrelvir/ritonavir, is an inhibitor of SARS-CoV-2 main protease. The potential for induction of viral resistance is discussed for each of these antivirals, along with their clinical activity on each of the SARS-CoV-2 variants and sublineages that have been dominant over the course of the pandemic, i.e., Alpha, Delta, as well as Omicron and its sublineages BA.1, BA.2, BA.5, BQ.1 and XBB. Host-directed antivirals are currently in preclinical or clinical development; these agents target host cell enzymes that are involved in facilitating viral entry, replication, or virion release. By blocking these enzymes, viral replication can theoretically be effectively stopped. As no SARS-CoV-2 host-directed antiviral has been approved so far, further research is still needed and we present the host-directed antivirals that are currently in the pipeline. Another specific type of agents that have been used in the treatment of COVID-19 are neutralizing antibodies (NAbs). Their main binding site is the spike protein, and therefore their neutralization activity is influenced by mutations occurring in this region. We discuss the main changes in neutralization activity of NAbs for the most important dominant SARS-CoV-2 variants. Close monitoring of emerging variants and sublineages is still warranted, to better understand the impact of viral mutations on the clinical efficiency of antivirals and neutralizing antibodies developed for the treatment of COVID-19.

## 1. Introduction

Severe acute respiratory syndrome coronavirus 2 (SARS-CoV-2) is a positive-sense single-stranded ribonucleic acid (RNA) betacoronavirus. RNA viruses generally lack the proofing mechanisms that are inherent to deoxyribonucleic acid (DNA) viruses, and are therefore considered to be more prone to frequent mutations, which can either lead to small inconsequential changes in the viral genome, or to multiple important changes that may characterize a new viral variant, which may present different transmission capacity, different virulence traits or different therapeutic susceptibility.

These evolutionary changes have been seen during the coronavirus disease-2019 (COVID-19) pandemic, when the accumulation of mutations in the viral genome led to the subsequent emergence of new viral variants. According to the World Health Organization (WHO)’s working definition, in order to consider a new SARS-CoV-2 strain to be a “variant of concern” (VOC), it should associate at least one of the following: Increase in transmissibility or detrimental change in COVID-19 epidemiology; OR Increase in virulence or change in clinical disease presentation; OR Decrease in effectiveness of public health and social measures or available diagnostics, vaccines, therapeutics” (World Health Organization, 2023).

This definition was met several times over the course of the pandemic. The initial wild type strain from December 2019, now referred to as the “index virus” as recommended by the WHO (World Health Organization, 2023), was completely replaced by the Alpha strain 1 year later, in December 2020. The Alpha VOC was, in turn, replaced in circulation by the Delta VOC in late summer and early autumn of 2021, and Delta was also relatively quickly replaced by the Omicron variant in December 2021, which has since remained dominant, while continuing to accrue mutations that led to the characterization of many sublineages.

With the prolonged dominance of Omicron, marked intra-VOC evolution has been noted, and the WHO has now provided a new working definition, for “Omicron subvariants under monitoring,” specifically that the sublineage should belong to a currently circulating VOC, and it should also “show signals of transmission advantage compared to other circulating VOC lineages; AND have additional amino acid changes that are known or suspected to confer the observed change in epidemiology and fitness advantage as compared to other circulating variants” (World Health Organization, 2023). The following Omicron sublineages have subsequently become dominant since December 2021: BA.1, BA.2, BA.5 and, more recently BQ.1, BQ.1.1, XBB and XBB.1.

Herein, we set out to describe the virological characteristics of the main SARS-CoV-2 VOCs and sublineages that have been dominant over the course of the pandemic, and to analyze their impact on the currently available treatment options. Furthermore, we also describe the clinically significant therapeutic developments that are in the pipeline by providing an in-depth review of the molecular mechanism of action of SARS-CoV-2 antivirals and critically analyzing the potential targets that may allow the selection of resistant viral variants.

## 2. SARS-CoV-2 variants of concern—Impact in clinical practice

Since the initial recognition as a novel coronavirus in early January 2020 (Wu et al., 2020), SARS-CoV-2 has been responsible for multiple infection waves characterized by high case counts, important morbidity and non-negligible mortality. Throughout the first 2 years of the pandemic, each wave was dominated by a new VOC that relatively rapidly replaced previously circulating variants from circulation.

Viral variant replacement has an important impact in clinical practice, as new variants may associate changes in transmissibility, severity of disease, and escape from pre-existing immunity (either naturally-acquired or vaccine-induced). With the exception of the Delta variant which, compared to previous and subsequent variants, was much more virulent and lead to higher rates of pneumonia, hospitalization and death (Taylor et al., 2021; Goncalves et al., 2022), the newly dominating Omicron variant and its sublineages appear to display lower pathogenicity, and lower occurrence of pneumonia or requirement for hospitalization (Goncalves et al., 2022). However, they also display higher infectivity and more pronounced evasion from pre-existing immunity. Despite a growing body of evidence regarding the virulence and clinical outcomes of novel dominant variants, there is still a gap in current knowledge regarding the exact impact that viral variant change has on the efficiency of available treatment options, as some are more likely to be prone to resistance if mutations occur with a higher frequency in the drug’s binding site. Herein, we explore the currently available treatment options, as well as the most important agents still in development, and we describe the potential resistance risk for each type of agent with the emerging viral variants and sublineages.

## 3. Antiviral agents

**Figure 1:**
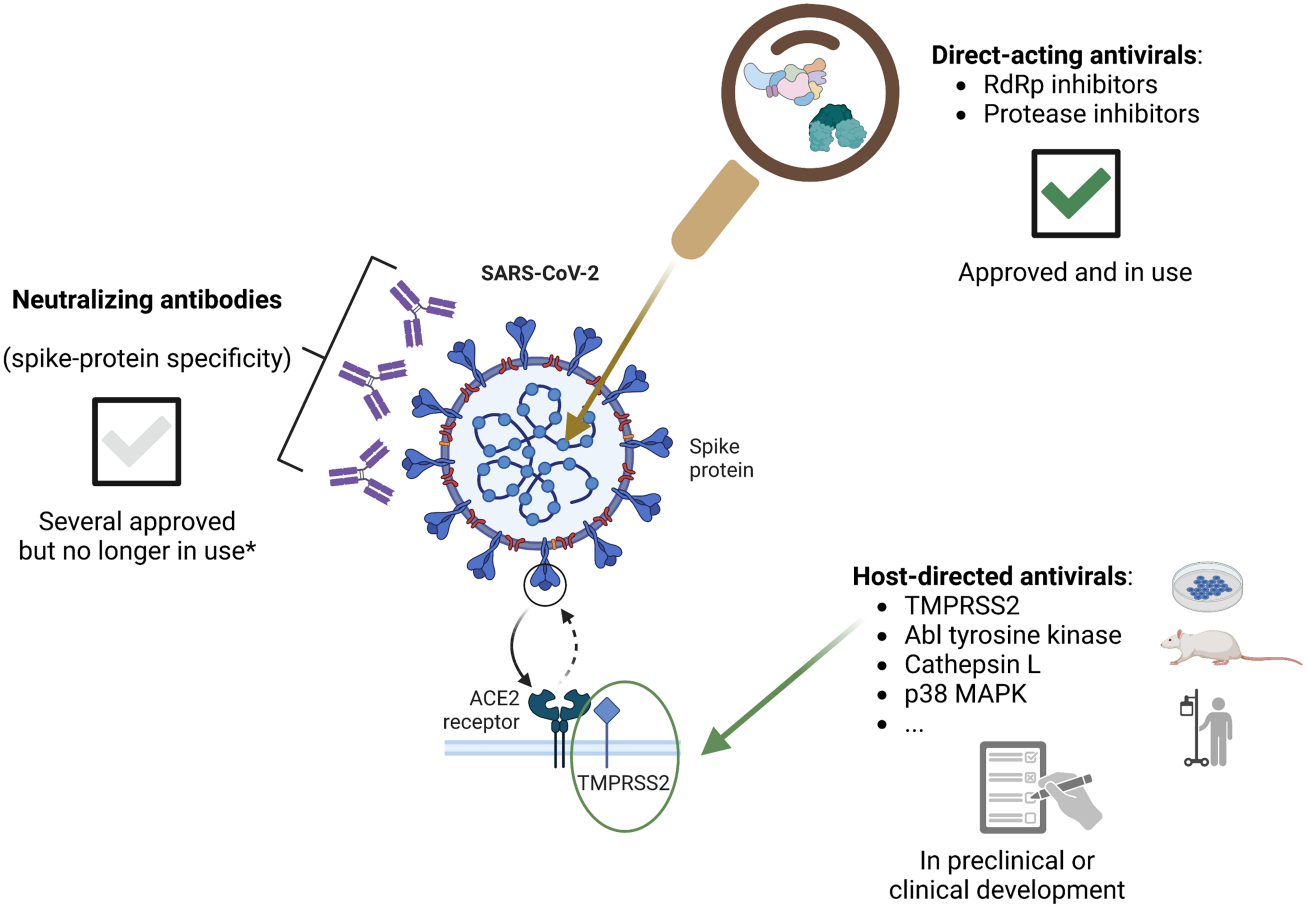
Classes of agents displaying antiviral activity against SARS-CoV-2, currently approved or in development (figure created with BioRender.com). *With the recent dominance of the BQ.1 and BQ.1.1 Omicron sublineages, as well as the emergence of Omicron XBB, all of the NAbs previously approved in the EU are considered to have lost neutralizing activity against the circulating variants in the region. However, this may change in the future should novel variants continue to emerge; furthermore, novel generation NAbs are still in development and some may prove to be effective.

Two main categories of agents can display antiviral activity. On the one hand, direct-acting antivirals act by inhibiting certain viral enzymes or other important viral components involved in the viral life cycle and thus stopping the replication of the virus. On the other hand, an antiviral effect can also be exhibited by drugs that target host cell factors that play a role in viral entry, replication, or virion release. Examples of agents that have been approved or are currently in development, from both these classes, are described below and depicted in Figure 1.

### 3.1. RNA-dependent RNA polymerase (RdRp) inhibitors

The RNA-dependent RNA polymerase (RdRp) is the 12^th^ among the 16 non-structural proteins (nsp) coded within the SARS-CoV-2 genome. It is also called nsp12, and it represents one of the core components of the viral replication and transcription complex. By itself, nsp12 shows limited enzymatic activity. In order to perform its biological polymerase functions, nsp12 requires the presence of other non-structural proteins that act as cofactors, such as nsp7 and nsp8. Together, they form the nsp12-nsp7-nsp8 complex, which represents the core component for viral RNA replication (Jiang et al., 2021). Two of the currently available antivirals act by inhibiting RdRp, specifically remdesivir and molnupiravir. Their mechanisms of action are described below.

#### 3.1.1. Remdesivir

Remdesivir was the first antiviral agent to receive approval for the treatment of COVID-19. It had been previously developed as part of the response to the Ebola epidemics in Africa, and rapid testing ascertained that it displayed antiviral activity against coronaviruses, including SARS-CoV-2, by inhibiting the RdRp. This antiviral effect is mediated mainly through its adenosine triphosphate (ATP) analogue activity. During SARS-CoV-2 replication, the phosphorylated form of the drug (remdesivir triphosphate – RTP) competes with cellular ATP and is preferentially incorporated as remdesivir monophosphate (RMP) into nascent viral RNA, which leads to delayed chain termination and disruption of further viral replication after the incorporation of three more nucleotides, phenomenon called “delayed termination” or “RdRp stalling” (Tchesnokov et al., 2020; Kokic et al., 2021). Furthermore, an additional mechanism of action is the promotion of polymerase read-through, i.e., paradoxical continuation of RNA synthesis past stop codons, to yield the full-length RNA product (Tchesnokov et al., 2020). This phenomenon occurs in the intracellular presence of increased nucleotide concentrations, such as those seen under normal conditions in host cells, and leads to the incorporation into the newly synthesized RNA copy of several remdesivir residues, which further inhibit the efficiency of incorporation of the complementary nucleotides (Tchesnokov et al., 2020).

The current approved indication for the use of remdesivir in the EU is the treatment of COVID-19 in adults and children (≥4 weeks of age and weighing ≥3 kg) who either have pneumonia requiring non-invasive supplemental oxygen (with a duration of treatment of 5 days, up to a maximum of 10 days), or who do not require supplemental oxygen but are at increased risk of progressing to severe COVID-19 (with a duration of treatment of 3 days). It is administered once daily by intravenous infusion, with an infusion duration of 30–120 min, established based on the dose (the loading dose on the first day requires a longer administration duration) and the infusion solution total volume.

#### 3.1.2. Molnupiravir

Molnupiravir is also a ribonucleoside analogue inhibitor of RdRp; it had been previously developed and studied for the treatment of influenza. Its active form, β-D-N4-hydroxycytidine triphosphate (NHC-TP) is preferentially used as substrate by SARS-CoV-2 RdRp, and incorporated instead of cytidine or uridine into the newly synthesized viral RNA. Compared to remdesivir, which terminates viral synthesis, molnupiravir allows the continuation of the process, but the resultant RNA template contains multiple mutations, with interconversions of cytidine to uridine and guanosine to adenosine, which cumulate to constitute catastrophic errors in the new virions, leading to formation of non-functional viruses (Yip et al., 2022; Streinu-Cercel et al., 2022a).

Molnupiravir’s use for the treatment of COVID-19 has been endorsed after Article 5(3) review by the European Medicines Agency (EMA) with the indication for use in adults who do not require supplemental oxygen and who are at increased risk of progressing to severe COVID-19 (Conditions of use, 2021). It is administered orally, twice daily, for 5 days, and is contraindicated during pregnancy and lactation.

#### 3.1.3. Antiviral activity of RdRp inhibitors across SARS-CoV-2 variants and sublineages

The RdRp is considered to be highly conserved across viral variants (Vangeel et al., 2022), and RdRp inhibitors have thus far displayed a low potential for the development of antiviral resistance. While mutations do occur in the RdRp region (Table 1) most do not appear to hinder the antiviral activity of the two drugs currently in use for the treatment of COVID-19. According to Stanford University Coronavirus Antiviral & Resistance Database, a list of 12 RdRp resistance mutations meet at least one of the following criteria and are currently being followed closely: they associate a ≥ 2.5-fold reduction in susceptibility to an RdRp inhibitor, and/or there is evidence of *in vitro* or *in vivo* selection of the mutation by an RdRp inhibitor in two or more studies or two or more patients receiving an RdRp inhibitor, respectively. This list includes the following mutations: R285C, A449V, D484Y, V557L, S759A, V792I, E796G, C799F, C799R, E802A, E802D, M924R (Tzou et al., 2022; Stanford University, 2022c).

**Table 1:** Mutations occurring in the RNA-dependent RNA polymerase (RdRp) region and main protease (M^pro^) region in the main dominant variants* since the beginning of the pandemic, and *in vitro* impact on antiviral activity.

Variant	Mutations in the RdRp region	Impact on remdesivir *in vitro* activity	Impact on molnupiravir *in vitro* activity	Mutations in the M^pro^ region	Impact on nirmatrelvir *in vitro* activity
Alpha	P323L	No significant change; Vangeel et al. (2022)	No significant change; Vangeel et al. (2022)		No significant change; Vangeel et al. (2022)
Delta	P323L, G671S	No significant change; Vangeel et al. (2022)	No significant change; Vangeel et al. (2022)		No significant change; Vangeel et al. (2022)
Omicron BA.1	P323L	No significant change (Vangeel et al., 2022; Veklury, 2022)	No significant change (Vangeel et al., 2022; Uraki et al., 2022b)	P132H	No significant change (Joyce et al., 2022; Vangeel et al., 2022)
Omicron BA.2	P323L	No significant change (Veklury, 2022; Takashita et al., 2022a)	No significant change (Takashita et al., 2022a; Uraki et al., 2022a)	P132H	No significant change (Takashita et al., 2022a; Uraki et al., 2022a)
Omicron BA.5	P323L	No significant change (Veklury, 2022; Takashita et al., 2022b)	No significant change (Takashita et al., 2022b)	P132H	No significant change (Takashita et al., 2022b)
Omicron BQ.1.1	Y273H, P323L	No significant change (Imai et al., 2022)	No significant change (Imai et al., 2022)	P132H	No significant change (Imai et al., 2022)
Omicron XBB	P323L, G671S	No significant change (Imai et al., 2022)	No significant change (Imai et al., 2022)	P132H	No significant change (Imai et al., 2022)

*Mutations are presented compared to the index Wuhan-HU-1 strain, according to Stanford University Coronavirus Antiviral & Resistance Database (Stanford University, 2022a).

When studying RdRp inhibitor activity in relation to variant change, both remdesivir and molnupiravir have continued to show antiviral activity across all VOCs that have become dominant over the course of the pandemic, with *in vitro* studies showing no significant change in activity for the Alpha, Beta, Delta and Omicron variants (Vangeel et al., 2022). When looking specifically at Omicron sublineages, remdesivir retained its activity across all major circulating sublineages, i.e., BA.1, BA.2, BA.4 and BA.5 (Veklury, 2022), as well as, most recently, BQ.1.1 and XBB (Imai et al., 2022). The same is true for molnupiravir, which has retained its antiviral efficacy against BA.1 (Uraki et al., 2022b) and BA.2 *in vitro* (Takashita et al., 2022a) and in hamster models (Uraki et al., 2022a), and *in vitro* against BA.5 (Takashita et al., 2022b), BQ.1.1 and XBB (Imai et al., 2022).

### 3.2. Protease inhibitor—Nirmatrelvir/ritonavir

The non-structural protein 5 (nsp5) of SARS-CoV-2 performs the activity of viral main protease (M^pro^), and is also called the 3C-like protease (3CLpro). Is it a cysteine hydrolase which is highly conserved among beta-coronaviruses. It plays an important role in viral replication: together with nsp3 (papain-like protease – PLpro), it cleaves the two main polyproteins that result from early translation of viral RNA, into the 16 non-structural proteins that are essential for the viral life cycle (Meyer et al., 2021).

Nirmatrelvir is an orally bioavailable peptidomimetic inhibitor of M^pro^. To exert its activity, it binds directly to the active site of M^pro^, and thus stops viral replication by blocking the processing of the polyprotein precursors. It is approved for the treatment of adults with COVID-19 who are at increased risk of progressing to severe disease and who do not require supplemental oxygen. Nirmatrelvir is co-administered with ritonavir, which acts as a pharmacological booster by inhibiting cytochrome P450 3A4. For this reason, drug–drug interactions should be carefully checked for each patient. Nirmatrelvir/r is administered orally, twice-daily, for 5 days.

Treatment-emergent amino acid substitutions in the M^pro^ region have been described to occur in cell cultures, and in clinical trials. The USA Food and Drug Administration (FDA) has particularly highlighted the E166V substitution, which emerged in nirmatrelvir’s binding site in M^pro^ in 3 (1%) of the nirmatrelvir recipients in the pivotal EPIC-HR clinical trial, and which has been shown *in vitro* to reduce the drug’s antiviral activity. However, there currently is no data to confirm or deny whether these substitutions have an impact on clinical efficacy (USA Food and Drug Administration, 2022). According to Stanford University Coronavirus Antiviral & Resistance Database, a list of 61 M^pro^ resistance mutations have a global prevalence >1/10000000 and are currently being followed (Tzou et al., 2022; Stanford University, 2022b).

From the data available so far, nirmatrelvir has retained antiviral activity across the most important dominant variants: Alpha, Delta (Vangeel et al., 2022), Omicron BA.1 (Joyce et al., 2022; Vangeel et al., 2022), BA.2 (Takashita et al., 2022a; Uraki et al., 2022a), BA.5 (Takashita et al., 2022b), BQ.1.1 and XBB (Imai et al., 2022).

### 3.3. Other antiviral agents in the pipeline

Much progress has already been made in the field of antiviral development for the treatment of SARS-CoV-2. However, many unmet needs still remain in the clinic and therefore, the drug discovery and testing process should be continued.

A search of clinicaltrials.gov performed in December 2022 for interventional trials with the indication “COVID-19” retrieved 1,554 trials currently being planned or already recruiting. Two of our authors screened these records to identify trials that met the following inclusion criteria: (1) studied antiviral candidates currently in clinical development, (2) for the treatment of SARS-CoV-2 infection, and (3) for which the proposed mechanism of antiviral action had been described in field literature. We excluded trials that: (1) studied non-pharmaceutical interventions, (2) studied already approved agents, (3) were indicated for COVID-19 prophylaxis only, or (4) lacked a scientific description of a potential antiviral mechanism. In Table 2, we present the main identified compounds, grouped by target of action for their antiviral effect.

**Table 2:** SARS-CoV-2 antiviral agents in the pipeline.

Drug target	Drug name	Clinical trial phase and planned period	Indication	Reference	Route of administration
Direct-acting antivirals					
SARS-CoV-2 RNA-dependent RNA polymerase	GS-5245 (oral formulation of remdesivir)	Phase 3, currently recruiting	Adults with COVID-19 at high risk of serious/severe illness	NCT05603143	Orally, 350 mg twice daily
	ASC10, prodrug of molnupiravir	Phase 1 in China, not yet recruiting	Patients with mild-to-moderate COVID-19	NCT05596045	Orally
	Azvudine	Phase not specified, recruiting	High-risk patients with COVID-19	NCT05642910	Orally
SARS-CoV-2 main protease	Ensitrelvir fumaric acid (S-217622)	Phase III, currently recruiting	Non-hospitalized patients with COVID-19	NCT05305547	Once daily, orally
		Phase III, not yet recruiting	Hospitalized patients with COVID-19	NCT05605093	Once daily, orally
	RAY1216	Phase III, not yet recruiting	Patients with mild-to-moderate COVID	NCT05620160	Orally
	SIM0417/ritonavir	Phase II, III, not yet recruiting	Adults with mild/common COVID-19	NCT05373433	Orally
		Phase II, III, not yet recruiting	Adults with mild/moderate COVID-19	NCT05506176	Orally
		Phase II, not yet recruiting	Adults with COVID-19	NCT05373446	Orally
	FB2001	Phase II, III, not yet recruiting	Hospitalized patients with moderate to severe COVID-19	NCT05445934	Twice daily intravenous infusion, 5 days
	GST-HG171/ritonavir	Phase II, III, not yet recruiting	Patients with mild-to-moderate COVID-19	NCT05656443	Orally
	PBI-0451	Phase II, currently recruiting	Non-hospitalized symptomatic adults with COVID-19	NCT05543707	Orally
	EDP-235	Phase II, currently recruiting	Non-hospitalized adults with mild or moderate COVID-19	NCT05616728	Orally
	STI-1558	Phase I, currently recruiting	Healthy volunteers	NCT05523739	Orally
Host-directed antivirals					
Transmembrane serine protease 2	Nafamostat mesylate	Phase I, II, recruiting	Patients with COVID-19	NCT04473053	Continuous intravenous infusion for 7 days
		Phase II, III, currently recruiting	Patients hospitalized with COVID-19	NCT04352400	Continuous intravenous infusion
	Camostat mesylate (DWJ1248)	Phase III, currently recruiting	Patients with severe COVID-19	NCT04713176	Orally, three times daily, up to 14 days, in association with remdesivir
Abl tyrosine kinase	Imatinib	Phase II, currently recruiting	Patients with COVID-19 receiving mechanical ventilation	NCT04794088	Intravenous infusion
Cathepsin L	SLV213	Phase II, not yet recruiting	Outpatients with COVID-19	NCT04843787	Orally
p38 mitogen-activated protein kinase	KIN001	Phase II, currently recruiting	Non-hospitalized patients with COVID-19	NCT05659459	Orally
Host translation cofactor eEF1A	Plitidepsin	Phase III, currently recruiting	Patients hospitalized for moderate COVID-19	NCT04784559	Intravenous infusion
Host IRE1α RNase endoplasmic reticulum stress response; interferon signaling pathways	Cannabidiol	Phase I, currently recruiting in Israel	Patients hospitalized for non-critical COVID-19	NCT04686539	Sublingual oil drops

Agents listed in the table were identified based on a search on clinicaltrials.gov performed in December 2022 for interventional trials with the indication “treatment of COVID-19.” The following inclusion criteria were applied: trials that (1) studied antiviral candidates currently in clinical development, (2) for the treatment of SARS-CoV-2 infection, and (3) for which the proposed mechanism of antiviral action had been described in field literature. We excluded trials that: (1) studied non-pharmaceutical interventions, (2) studied already approved agents, (3) were indicated for COVID-19 prophylaxis only, or (4) lacked a scientific description of a potential antiviral mechanism.

#### 3.3.1. Direct-acting antivirals

The two main classes of direct-acting antivirals for SARS-CoV-2 include RdRp inhibitors and main protease inhibitors.

Among RdRp inhibitors, we found three listed agents, all with oral bioavailability. Of these, two compounds were not actually novel agents, but an oral formulation of remdesivir and a different prodrug of molnupiravir. The third identified agent, azvudine (2′-deoxy-2′-β-fluoro-4′-azidocytidine – FNC), is a nucleoside analog used locally in China for the treatment of HIV infection, which also has been reported to display an antiviral effect on SARS-CoV-2, through inhibition of RdRp (Ren et al., 2020; Zhang et al., 2021). It has recently received regulatory approval in China in 2022 for the treatment of COVID-19 but phase III local clinical trial data have not yet been communicated. Further clinical trials are listed on clinicaltrials.gov as recruiting in China, aiming to assess the efficacy of azvudine compared to nirmatrelvir/ritonavir in high-risk patients with COVID-19.

Among SARS-CoV-2 main protease inhibitors, we have identified 8 compounds that are currently being evaluated, of which 7 have oral bioavailability and 1 requires intravenous administration. Among these main protease inhibitors currently in the pipeline, ensitrelvir fumaric acid appears to be the most advanced; it is administered orally once daily for 5 days. It demonstrated rapid decrease in SARS-CoV-2 viral titers in a phase II clinical trial (Mukae et al., 2022), and has also been announced to have achieved its primary endpoint, median time to resolution of five main COVID-19 symptoms, in a phase III trial in patients with mild-to-moderate COVID-19, of which most patients had been previously vaccinated; other phase II trials are ongoing (Table 2).

#### 3.3.2. Host-directed antivirals

Agents that target the transmembrane serine protease 2 (TMPRSS2) are able to inhibit viral entry of SARS-CoV-2, and examples include camostat mesylate or nafamostat mesylate, described below.

Camostat mesylate is an oral SARS-CoV-2 TMPRSS2 inhibitor that showed a good safety profile but no statistically significant reduction in time to clinical improvement in phase II clinical trials for patients with mild-to-moderate COVID-19 (Kim Y S, et al., 2022). It is currently assessed in phase III clinical trials for the indication of severe COVID-19 (Table 2).

Nafamostat mesylate is also an inhibitor of TMPRSS2, which has been assessed in patients hospitalized with COVID-19 pneumonia requiring nasal high-flow oxygen therapy or non-invasive mechanical ventilation. The results of the phase II trial showed no significant difference in the overall time to clinical improvement, but with a trend towards a shorter median duration to clinical improvement in a small group of high-risk patients requiring oxygen treatment, i.e., those with a baseline NEWS score ≥ 7 and not receiving corticosteroid therapy (Zhuravel et al., 2021); this warranted continuation of clinical development into phase III clinical trials (Table 2).

Imatinib, an Abl tyrosine kinase inhibitor, has shown promising *in vitro* activity against SARS-CoV-2, inhibiting both types of viral entry, TMPRSS2-dependent and TMPRSS2-independent, i.e., membrane-fusion and endocytosis (Strobelt et al., 2022). It is now undergoing assessment in a phase II clinical trial in patients with COVID-19 receiving mechanical ventilation.

Other host-directed antivirals in the pipeline include those targeting human host cell cysteine proteases such as cathepsin L (SLV213), MEK1/2 kinases inhibitors (zapnometinib; Schreiber et al., 2022), small molecule inhibitors of p38 mitogen-activated protein kinase (p38 MAPK), host translation cofactor eEF1A (plitidepsin; Martinez, 2021), up-regulators of the host IRE1α RNase endoplasmic reticulum stress response and interferon signaling pathways (cannabidiol – CBD; Nguyen et al., 2022), for which a phase 1 clinical trial is currently recruiting in Israel (Tzou et al., 2022).

## 4. Neutralizing anti-spike protein antibodies

The development of therapeutic and prophylactic anti-spike protein neutralizing antibodies (NAbs), also called monoclonal antibodies (mAbs), started shortly after the beginning of the pandemic, through the identification of naturally occurring highly neutralizing antibodies from patients convalescing from COVID-19. The first NAbs clinical trials started during the second and third COVID-19 waves, throughout the first important variant change, when Alpha became dominant internationally in late 2020, leading to the approval in November 2021 by the EMA of the first SARS-CoV-2 anti-spike protein NAbs, based on the data from the pivotal phase III trials, casirivimab/imdevimab (O’Brien et al., 2021, 2022) and regdanvimab (Kim et al., 2021; Kim J Y, et al., 2022; Streinu-Cercel et al., 2022c), followed, 1 month later, in December 2021, by sotrovimab (Heo, 2022), and in March 2022 by tixagevimab/cilgavimab (Keam, 2022).

In the United States, a set of other NAbs have also received FDA approval over the course of the pandemic, including bamlanivimab/etesevimab and bebtelovimab, and several other NAbs have applied for FDA review. However, the recommendation for the use of different approved NAbs was periodically revised with each variant change. This was of utmost importance because the replacement of the dominant variant was due to important changes in the spike protein, particularly in the receptor-binding domain (RBD), which is the main target of most NAbs. While some minor changes were associated with limited decreases in the neutralization potency of the NAb, major changes led to loss of activity of many of the previously approved NAbs. This is not “resistance” *per se*, but rather reduced neutralization activity due to decreased binding of the NAbs to their targets on the viral spike protein. However, this decrease in neutralization has rendered many of the NAbs clinically ineffective.

The first important variant change since the approval of many NAbs was the dominance of the Delta VOC starting with summer/autumn 2021. Fortunately, most approved NAbs retained their clinical efficacy and continued to be successfully used throughout the wave that had the highest severity and highest occurrence of pneumonia up until that point in the pandemic (Taylor et al., 2021; Goncalves et al., 2022; Miguez-Rey et al., 2022). Specifically, only bamlanivimab suffered from a > 1,000-fold decrease in neutralization activity for Delta and its approval as single agent was revoked, while it continued to be clinically effective and approved for use when coadministered with etesevimab (Tzou et al., 2022; Stanford University, 2022d). To reduce the burden on the hospitals, outpatient departments, also called “infusion clinics,” as well as mobile units were created (Ton et al., 2021; Tulledge-Scheitel et al., 2021). Here, patients at high-risk for progression to severe COVID-19 were treated with NAbs as early as possible after symptom onset, with good clinical response and lower rates of hospitalization or requirement for supplemental oxygen (Al-Obaidi et al., 2022; Kwak et al., 2022; Stan et al., 2022). At that point in the pandemic, antivirals were considered “rescue therapy” and used only in cases where NAbs had either not been administered, or in cases of progression to pneumonia despite NAb treatment. For example, in Romania, the only SARS-CoV-2 specific antiviral available at that point (autumn 2021) was remdesivir, and it was still being used exclusively for patients with severe disease, according to its on-label indication at that time.

The next variant change saw the dominance of the Omicron variant starting with December 2021. This was in fact the first major change impacting NAbs neutralization activity, rendering most NAbs clinically ineffective, with the exception of sotrovimab which retained activity against omicron BA.1, and bebtelovimab, which subsequently received emergency use authorization in February 2022 (Tzou et al., 2022; Stanford University, 2022d). The neutralization activity differed for different Omicron sublineages. For example, NAbs that were no longer active on BA.1, which was the first dominant sublineage, regained some high-dose activity against BA.2, i.e., casirivimab/imdevimab and tixagevimab/cilgavimab (Takashita et al., 2022a). However, this gain in activity was short-lived, as the BA.2 sublineage was rapidly replaced by BA.5, against which bebtelovimab was the only NAb to retain neutralization activity (Tzou et al., 2022; Stanford University, 2022d), while tixagevimab/cilgavimab had a 10-100-fold reduced activity but continued to be considered by the Infectious Diseases Society of America (IDSA) for pre-exposure prophylaxis for patients with moderate or severe immunodepression with inadequate immune response to COVID-19 vaccination (Bhimraj et al., 2022).

The initial Omicron period in late 2021 and early 2022 was when SARS-CoV-2 antivirals started to be used increasingly early, as novel agents become available, with the advice for early use of molnupiravir in outpatients with COVID-19 followed in December 2021 by the expanded indication of remdesivir to adult patients who are not on supplemental oxygen but considered at high risk of progression to severe COVID-19, and the subsequent approval of nirmatrelvir/r.

With the recent dominance of the BQ.1 and BQ.1.1 Omicron sublineages, as well as the emergence of Omicron XBB, all of the previously approved NAbs display markedly decreased neutralization activity (Tzou et al., 2022; Stanford University, 2022d) and in the beginning of December 2022 the EMA Emergency Task Force provided a statement that although no clear correlates have been established between *in vitro* activity, achievable plasma or tissue NAb concentrations, and clinical efficacy, it is now considered that all of the currently approved NAbs in the EU have lost activity against the circulating variants in the EU, and that antivirals should be used instead in patients at high-risk of COVID-19 progression. The IDSA has also revised the phrasing of the statement regarding NAbs, and instead of listing all different NAbs, it now simply suggests “treatment with anti-SARS-CoV-2 monoclonal antibodies with activity against the predominant regional variants” (Bhimraj et al., 2022).

## 5. Discussion

The current COVID-19 pandemic has taught us important lessons about evolutionary virology and particularly about the important role that molecular genetics play in informing and guiding clinical practice. With the viral evolution of SARS-CoV-2, each variant replacement came silently, with no specific signs or symptoms that could aid in differentiating viral variants based on clinical observation alone (Streinu-Cercel et al., 2022b). Each of the dominating variants showed increased transmissibility but different virulence and pathogenicity, traits which were also influenced by COVID-19 vaccination, which became available in late December 2020. More importantly, the emergence of new dominant viral variants has significantly impacted the clinical efficacy of different treatment options, highlighting the importance of rapid molecular diagnosis in choosing evidence-based treatment, particularly during variant changes.

So far, direct-acting antivirals such as RdRp and M^pro^ inhibitors have retained activity across all dominant variants and sublineages, despite the occasional occurrence of mutations in the RdRp and M^pro^ genomic regions. The occurrence of these mutations is being closely monitored, and so is the antiviral efficacy of the approved agents; so far, no signals of major concern have arisen.

This was not the case for NAbs, whose neutralization activity was significantly influenced by mutations occurring in the spike region. While many NAbs are still in preclinical and clinical development, if mutations in the spike region continue to occur at the current rate, it is becoming unlikely that novel agents will reach clinical practice in time, before the emergence of a novel variant with potentially decreased binding affinity. However, while novel sublineages have emerged at a faster pace than before, so far in 2022 we have not seen a completely novel variant becoming dominant, but rather sublineages derived from Omicron predecessors. For example, the currently dominant BQ.1 is a sublineage of BA.5, while the emerging XBB is a sublineage of BA.2, representing a recombinant of the BA.2.10.1 and BA.2.75 sublineages. Should this remain the case, that sublineages of the Omicron variant subsequently replace each other, without the emergence of a significantly different variant, this could indicate that novel generation NAbs currently in development might be less affected by the antigenic variations between sublineages and could retain, to a certain extent, their neutralization activity, depending on the frequency and the nature of the cumulated mutations in the spike region.

There is yet much to learn in terms of viral evolution of SARS-CoV-2 and how to best make use of the available therapeutic instruments to provide evidence-based treatment recommendations, and to translate these into personalized treatment decisions for each patient. The SARS-CoV-2 antiviral pipeline holds promise for the future armamentarium for COVID-19, however, there are still important unmet needs in the clinic.

## 6. Conclusion

The therapeutic options for COVID-19 have evolved in parallel with the changing landscape of the disease, each subsequent COVID-19 wave bringing its own therapeutic challenges. While first generation anti-spike protein neutralizing antibodies were developed relatively quickly, tested during the Alpha wave and used extensively in the clinic during the Delta wave, the dominant Omicron variant has significantly impacted their neutralization activity, and all previously approved agents have been rendered ineffective for the emerging BQ.1 and XBB Omicron sublineages, while some remain in clinical development. Treatment is now focused on direct-acting antivirals, currently approved agents being inhibitors of the RdRp or the main protease. These have retained their efficacy across all subsequently dominant variants, and across Omicron sublineages, and they continue to play an important role in current clinical practice. Furthermore, a set of direct-acting antivirals are still in clinical development, and so are host-directed antivirals, that target host cell enzymes in order to block viral replication. Close monitoring of emerging variants and sublineages is still warranted, to better understand the impact of viral mutations on the clinical efficiency of antivirals and neutralizing antibodies developed for the treatment of COVID-19.

## Author contributions

OS, CGA, LLP, AS-C, and MS contributed equally to the manuscript in conceptualization, data curation, writing—original draft, writing—review and editing, visualization, and supervision. All authors contributed to the article and approved the submitted version.

## Conflict of interest

OS has been investigator in COVID-19 clinical trials by Algernon Pharmaceuticals, Atea Pharmaceuticals, Diffusion Pharmaceuticals, Regeneron Pharmaceuticals, PureTech, Celltrion Inc., Adagio Therapeutics and Atriva Therapeutics. LLP has been investigator in COVID-19 clinical trials by Celltrion Inc., and PharmaMar. AS-C has been investigator in COVID-19 clinical trials by Algernon Pharmaceuticals, Atea Pharmaceuticals, Diffusion Pharmaceuticals, Regeneron Pharmaceuticals, PureTech, and Celltrion Inc.

The remaining authors declare that the research was conducted in the absence of any commercial or financial relationships that could be construed as a potential conflict of interest.

## Publisher’s note

All claims expressed in this article are solely those of the authors and do not necessarily represent those of their affiliated organizations, or those of the publisher, the editors and the reviewers. Any product that may be evaluated in this article, or claim that may be made by its manufacturer, is not guaranteed or endorsed by the publisher.
